# Immovable Object Meets Unstoppable Force? Dialogue Between Resident and Peripheral Myeloid Cells in the Inflamed Brain

**DOI:** 10.3389/fimmu.2020.600822

**Published:** 2020-12-08

**Authors:** Alanna G. Spiteri, Claire L. Wishart, Nicholas J. C. King

**Affiliations:** ^1^ Discipline of Pathology, Faculty of Medicine and Health, School of Medical Sciences, The University of Sydney, Sydney, NSW, Australia; ^2^ Charles Perkins Centre, The University of Sydney, Sydney, NSW, Australia; ^3^ Sydney Cytometry Facility, The University of Sydney and Centenary Institute, Sydney, NSW, Australia; ^4^ Ramaciotti Facility for Human Systems Biology, The University of Sydney and Centenary Institute, Sydney, NSW, Australia; ^5^ Marie Bashir Institute for Infectious Diseases and Biosecurity (MBI), Faculty of Medicine and Health, Sydney Medical School, The University of Sydney, Sydney, NSW, Australia; ^6^ Nano Institute, The University of Sydney, Sydney, NSW, Australia

**Keywords:** microglia, neuroinflammation, central nervous system infiltration, neuropathology, central nervous system infection, monocyte-macrophage

## Abstract

Inflammation of the brain parenchyma is characteristic of neurodegenerative, autoimmune, and neuroinflammatory diseases. During this process, microglia, which populate the embryonic brain and become a permanent sentinel myeloid population, are inexorably joined by peripherally derived monocytes, recruited by the central nervous system. These cells can quickly adopt a morphology and immunophenotype similar to microglia. Both microglia and monocytes have been implicated in inducing, enhancing, and/or maintaining immune-mediated pathology and thus disease progression in a number of neuropathologies. For many years, experimental and analytical systems have failed to differentiate resident microglia from peripherally derived myeloid cells accurately. This has impeded our understanding of their precise functions in, and contributions to, these diseases, and hampered the development of novel treatments that could target specific cell subsets. Over the past decade, microglia have been investigated more intensively in the context of neuroimmunological research, fostering the development of more precise experimental systems. In light of our rapidly growing understanding of these cells, we discuss the differential origins of microglia and peripherally derived myeloid cells in the inflamed brain, with an analysis of the problems resolving these cell types phenotypically and morphologically, and highlight recent developments enabling more precise identification.

## Introduction

Like other organs of the body, it is now well established that the central nervous system (CNS) has its own unique immune system that constantly maintains homeostasis and is rapidly engaged during inflammatory insult. Arguably, microglia are the key regulators of the immune response in the healthy brain. However, under certain conditions, such as those underlying neurodegenerative disease, autoimmunity, infectious encephalitis, and ischemia, infiltration of bone marrow (BM)-derived monocytes act in concert with local microglia in the brain parenchyma to initiate, enhance, or dampen immune activity. Resident and infiltrating myeloid cells in the inflamed brain may be developmentally distinct, but often adopt similar morphologies and phenotypes, complicating accurate identification. More nuanced tools have improved resolution, and through these we can better define populations in the brain, allowing further elucidation of the role of resident and peripherally infiltrating myeloid cells in the inflamed brain. Given the fast-developing field, and the evident importance of both microglia and BM-derived monocytes to disease processes in a variety of CNS pathologies, we review the current understanding of the origins and functions of these cell types in homeostasis and highlight new experimental tools, molecules, and drugs which may overcome issues of differentiating between these populations during neuroinflammation.

## Microglia Origins and Renewal

**Figure 1:**
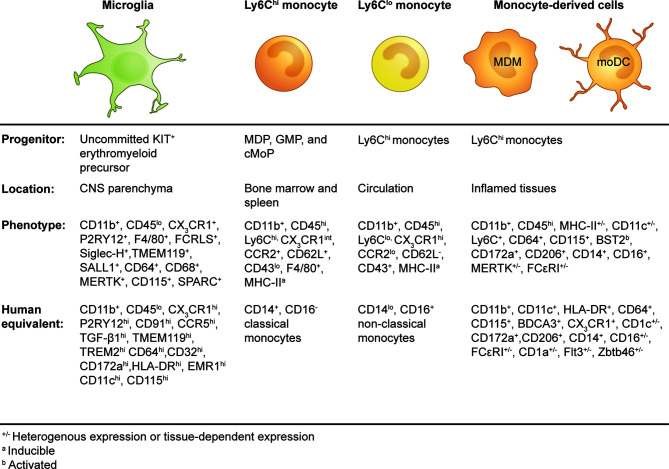
Origins and phenotypes of resident microglia and monocyte-derived cells in the periphery and inflamed brain.

Historically, microglia were first believed to be of neuroepithelial origin (1, 2), along with neurons and neuroglia. Subsequently, they were thought to be of monocytic origin (3), derived from hemopoietic stem cells (HSCs) in the fetal liver or BM. In 1999, Alloit et al. proposed the yolk sac (YS) origin of microglia (4). A decade later, this was confirmed using a fate-mapping model to trace YS progenitors, replacing the view of a monocytic origin for microglia (5). Microglia are now known to arise from uncommitted KIT^+^ erythromyeloid precursors (EMP) (6) (**Figure 1**), which seed the brain from the YS at embryonic day 9.5 (E9.5) in the mouse (5), well before other glial cells and before the formation of the blood-brain barrier (BBB) (6, 7). However, other evidence suggests that microglia are not exclusively YS-derived, and that a small population arise from Hoxb8^+^ progenitors in the E12.5 fetal liver (8) or from fetal HSC-derived monocytes (9). Subsequent to the formation of the brain, microglia are renewed *in-situ* throughout life, independently of BM-derived HSCs (10–12). In the steady state, microglia have region-specific renewal rates (13) with their density maintained *via* the tight coupling of apoptosis and proliferation (14). In the mouse brain, half the microglial population persists throughout the entire lifespan of the animal and thus remains a relic of the embryonic brain (11). In young and adult mice, the median life span of microglia is 22 and 29 months, respectively (11). In humans, microglia can survive for more than twenty years, although unlike mice, their entire population is renewed at a median rate of 28% per year (10). The correlation of high differential renewal rates with microglial function remain to be revealed.

The microglial phenotype is derived from the successive development of uncommitted KIT^+^ EMP into the macrophage ancestor population A1 (CD45^+^, CX3CR1^-^, F4/80^-^) and then into the A2 progenitor population (CD45^+^, CD115^+^, CX3CR1^+^, F4/80^hi^), which migrates to and populates the embryonic brain (6). The development of microglia from precursor cells into intermediate progenitors is a finely tuned process orchestrated by external and internal stimuli. PU.1, RUNX1, and IRF8 are indispensable transcription factors in the programming of EMP into microglia during embryonic development (5, 6, 15, 16). CD115 (also known as colony-stimulating factor-1 receptor, CSF-1R, or macrophage colony-stimulating factor receptor, M-CSFR) ligands, CSF-1, and interleukin (IL)-34, are important for the maintenance of microglia in the adult brain, with IL-34 being highly expressed by neurons in a region-specific manner in the adult mouse brain (17, 18). CD115 signaling is more critical during development, playing an important role in the differentiation of EMP into microglia, but is also required for replenishment of adult microglia and maintenance (18–20). CD115-deficient mice have reduced microglial numbers, and treatment with CD115 inhibitors at high doses results in significant microglial depletion (19, 21).

Remarkably, microglia rapidly renew their entire population after chemical or genetic (conditional) depletion. Depending on the depletion method, the presence of non-physiological perturbations, and/or the experimental model, studies have suggested this occurs through niche repopulation by infiltrating monocytes, proliferation of a microglial progenitor or proliferation of surviving microglia. In the absence of BBB breakdown or lethal irradiation and BM transplant, it is believed that there is little or no contribution of HSC-derived monocytes to the microglial pool (12, 22) and that surviving microglia repopulate *via* self -renewal (23–25). Experimental methods used to deplete myeloid cells in the CNS and periphery are presented in **Table 1**. In irradiated BM-reconstituted CD11b-herpes simplex virus thymidine kinase (HSV-TK) mice injected intracerebroventricularly (i.c.v.) with ganciclovir to deplete microglia, engrafted “microglia” were of peripheral origin (45) (**Table 1**). On the other hand, following treatment with PLX3397, a small-molecule CD115 inhibitor, replacement microglia arose from a resident microglial progenitor population expressing nestin, a neural stem cell marker that can also be expressed on macrophages (19) (**Table 1**). By contrast, microglial depletion in either Cx3cr1CreER:iDTR mice, in which long-lived CX3CR1^+^ cells (microglia) are depleted after tamoxifen and diphtheria toxin (DTx) administration (**Table 1**) (25) or with the CD115 inhibitor PLX5622 (24, 46), showed little contribution of nestin^+^ progenitors or peripheral myeloid cells to the regenerating microglial pool, supporting the innate capacity for microglial self-renewal (**Table 1**). The specific attributes required for microglial survival (and thus incomplete depletion) during these depletion procedures are unclear, but there is an implied refractoriness in the pathways involved in surviving microglia reminiscent of a developmental stage difference or “stemness,” with survivors clearly retaining the ability to proliferate for population renewal.

**Table 1:** Methods of myeloid cell blockade and depletion.

Depletion method	Cell type targeted by method of depletion	Drawbacks	Mechanism of action
**Intravenous administration of clodronate-encapsulated liposomes**	Circulating monocytes and phagocytic cells in the bone marrow, liver and spleen (26–28)	Depletion is incomplete Clodronate liposomes do not specifically deplete one monocyte/macrophage subset	If clodronate is administered *via* an intravenous injection, clodronate liposomes in homeostatic animals cannot leave the blood vessels unless through sinusoids, and are thus limited to the circulation, bone marrow, liver, and spleen. In inflammatory conditions where the endothelium allows extravasation of molecules, liposomes can pass through. Liposomes containing clodronate are engulfed by phagocytic cells. Once in the cell, liposomes fuse with lysosomes causing the disruption of liposome bilayers, which allows the intracellular release of clodronate. Clodronate above a threshold concentration, causes irreversible damage to the cell and subsequent apoptosis (29).
**Targeting chemokine receptor CCR2**	CCR2-expressing monocytes in the bone marrow	Cells are not depleted but blocked from entering the circulation and thus do not reach inflamed tissue. Using anti-CCL2 (CCR2 ligand) monoclonal antibody (mAb) results in incomplete blockade (30).	Intravenous (i.v.) or intraperitoneal (i.p.) injection of anti-CCR2 or CCL2 mAb (31) or by the use of transgenic CCR2^−/−^ mice (32). Monocytes are prevented from leaving the bone marrow via blocking the CCL2-CCR2 signaling axis.
**Intracerebroventricular (i.c.v.) administration of clodronate liposomes**	Microglia (33)	Invasive procedure which breaches the BBB Incomplete depletion	Clodronate liposomes are administered intracranially and engulfed by phagocytic cells in the brain, causing their “suicide” *via* apoptosis (26)
**Transgenic animals with mutations in genes critical for microglial development and maintenance:** PU.1, CD115 (CSF1R) and TGF-β	In CNS-TGFβ1^−/−^ mice (*i.e.* IL2*TGF-β1*-Tg-TGF-β1^−/−^: TGF-β1 is thus limited to T lymphocytes): Microglia (25) In CSF1R^−/−^: Microglia, monocytes and tissue resident macrophages (5) In PU.1^−/−^ mice: Microglia, mature myeloid cells and B cells (6)	These mice rarely survive into adulthood and develop defects in other organs other than the brain (34). Incomplete microglia depletion in CNS-TGFβ1^−/−^ mice and an increase in peripherally derived cells into the CNS (CD39^−^CD11b^hi^Ly6C^+^) (25)	Genes required for development and maintenance of microglia were genetically deleted, resulting in their depletion.
**CD11b-HSVTK mice**	Gamma-irradiation-resistant CD11b^+^ cells (*i.e.* microglia)	Incomplete bone marrow reconstitution and prolonged ganciclovir (GCV)-administration causes myelotoxicity and can be fatal (35). GCV administered orally or *via* an i.p. injection results in incomplete microglia depletion. Instead microglia proliferation and activation is blocked (35). Compromise of the BBB if GCV is administered i.c.v. and also extended application of GCV this way, causes microhemorrhages and influx of peripheral macrophages into the CNS (36).	Host mice express herpes-simplex virus thymidine kinase (HSV-TK) under the CD11b-promoter are lethally irradiated and engrafted with WT BM (35). Only irradiation resistant CD11b^+^ cells express HSV-TK. GCV administered *in-vivo* is converted into a monophosphorylated form *via* HSV-TK. Endogenous cellular kinases then convert the monophosphorylated form of GCV into a toxic triphosphate. GCV competes with thymine for DNA synthesis and thus preferentially targets proliferating cells. Non-proliferating cells have a reduced susceptibility to GCV. GCV administered orally or *via* an i.p. injection does not result in complete microglia depletion, but microglia “paralysis” whereby these cells are unable to proliferate or become “activated” (35). However, administrating GCV i.c.v. *via* an osmotic pump causes 90% depletion after two weeks (36).
**CX3CR1^CreER^DTR** **mice**	Long lived CX3CR1^+^ cells (microglia, and most likely BAMs)	Repopulation in 5 days (23) Incomplete depletion—20% of microglia remained (23). Although Parkhurst et al. (37) showed a 99% depletion rate. Astrogliosis and “massive” production of cytokine and chemokines (cytokine storm) (23). Mice showed impaired learning and dendritic spine elimination (37)	Mice expressing Cre-recombinase (Cre-ER) under the *CX3CR1* promoter were crossed with iDTR animals. Tamoxifen (TAM) administration causes the nuclear translocation of the CreER fusion protein resulting in cre-mediated recombination and the expression of the diphtheria toxin receptor (DTR) on CX3CR1^+^ cells. Nuclear translocation of the CreER fusion protein is transient and lost shortly after TAM treatment in short-lived CX3CR1^+^ cells that are readily renewed in the BM *via* HSC. Long-lived CX3CR1^+^ cells express DTR, thus after systemic administration of diphtheria toxin (DTx), which can pass through the BBB, these cells are ablated. This system does not require the generation of a BM chimera and thus avoids the non-physiological effects observed with whole body irradiation, including BBB disruption and peripheral immune cell infiltration into the CNS.
***Sall1*^CreER^*Csf1r*^fl/fl^** **mice**	Microglia	Incomplete microglia ablation. 70–90% of microglia are deleted in various brain regions (38). Requires mouse breeding and generation of transgenic animals. Tamoxifen may result in an immunomodulatory phenotype in mice (34).	TAM administration induces the nuclear translocation of the CreER fusion protein in Sall1^+^ cells. Cre-recombinase then drives the deletion of floxed *Csf1r*, causing the ablation of microglia (38). Sall1 is thought to be a microglia-specific marker, thus this depletion method is very specific to microglia.
Pharmacological inhibition of CD115 (CSF1R) using **PLX3397**	Microglia, HSC, osteoclasts, macrophages, and mast cells	Inhibits three other kinases including FLT3, PDGFR, and KIT (39–41). Repopulation once the drug is withdrawn Broad myelosuppression and astrogliosis (34).	PLX3397 is a CD115 (CSFIR) inhibitor that is typically formulating into a rodent chow and administered orally (19). CD115 signaling is required for microglial development and maintenance, thus inhibition of this receptor results in microglial ablation. Unlike all the other depletion methods listed above, microglia can be targeted without the breeding of transgenic animals, or the use of irradiation to achieve chimerism or the use of an invasive procedure which compromises the BBB. PLX3397, causes 50% microglia depletion within 3 days, and >99% depletion after 21 days of treatment (at 290 ppm) (19). At 75 ppm PLX3357 causes CSF1R inhibition without ablating microglia (21).
Pharmacological inhibition of CD115 (CSF1R) using **PLX5622**	Microglia	Rapid repopulation after the drug is withdrawn. Incomplete microglia depletion (21) Affects haemopoiesis and macrophage phenotype and function in the spleen, BM and blood (42–44)	PLX5622, like PLX3397 is a CD115 (CSFIR) inhibitor which is also typically formulated into a rodent chow to be administered orally. Both PLX3397 and PLX5622 have the same potency for inhibiting CD115. PLX5622, however, has a 20-fold selectivity for CD115 over other kinases (KIT and FLT3) and a ~15% increase in BBB penetrance (has a lower molecular weight, higher lipophilicity, and better cell permeability), compared to PLX3397 (21) and can yield 90% microglia depletion within 5 days (at 1,200 ppm in chow).

The concept that microglia are capable of self-renewal without input from peripheral myeloid cells, both in homeostasis and disease, was established in “microfetti” mice (*Cx3cr1* ^CreER^ mice crossed with R26RConfetti reporter mice) and in a model of parabiosis. In microfetti mice, replenished microglia are tagged with one of four reporter proteins of the confetti labelling system, giving information on the distribution, expansion, and clonality of repopulating microglia. After unilateral facial nerve axotomy, microglia underwent rapid self-renewal with no contribution from progenitors or external myeloid populations (13). In the parabiosis model, a transgenic mouse expressing green fluorescent protein (GFP) in hemopoietic mononuclear cells and a wild type (WT) mouse were surgically attached for several weeks to achieve 50% blood chimerism (12). When the WT mice were subjected to facial nerve axotomy or amyotrophic lateral sclerosis (ALS), the CNS of the WT mice had no GFP^+^ cells (partner-derived cells), demonstrating *in-situ* microglial repopulation.

While the capacity of microglia to self-renew without contribution from the periphery has emerged as the dogma, these ideas were established using parabiotic mice and mild inflammatory insults. During severe inflammatory insult and/or perturbation of the BBB, it was speculated that microglia could be derived from circulating peripheral monocytes (30). As the circulating myeloid compartment serves as a reservoir of immune cells that can rapidly be recruited to any tissue as needed, whether to contain virus or assist in tissue repair after traumatic injury, this may be an additional pragmatic solution to replenishing microglia, either in the short or long term, notwithstanding a likely differential genetic signature (47). It is unclear if such BM precursors are sufficiently stem-like to become “real” microglia once in the CNS, and if so, whether they could become a completely self-renewing immigrant population that can maintain a density and network configuration similar to native microglia. Whether such engrafted “microglia” would function similarly to YS-derived microglia in both homeostasis and pathology over time, is of considerable interest and still unresolved.

## A Day in the Life of Microglia: Functions in the Embryonic and Adult CNS

The importance of microglia to normal CNS development and homeostasis has been historically underappreciated. While microglia have long been recognized for their role as resident tissue macrophages, this extends considerably further than their innate immunological “first-line of defense” functions.

### Embryonic Brain

As microglia seed the brain during early embryogenesis, they display an “activated,” ameboid morphology as they proliferate and migrate throughout the CNS (48, 49). Upon CNS maturation, microglia become more sessile and adopt a highly ramified morphology (49). The importance of microglia to embryonic development in the CNS has been shown in several depletion models, with ablation of these cells causing long term effects on normal brain functioning. For example, the absence of embryonic microglial progenitors caused defects in dopamine innervation and cortical networks (50), whilst neuronal survival was reduced in CX3CR1-deficient and microglia-ablated CD11b-DTR mice, arguably from the absence of CX3CR1-dependent production of neurotrophic insulin-like growth factor-1 (IGF-1) (51). Absence of microglia in mice homozygous for the null mutation in the CSF-1 receptor (*Csf1r^−/−^*) revealed a disruption to brain morphology and neuronal density, as well as significantly affecting total astrocyte and oligodendrocyte numbers (52). Further, depletion of microglia using PLX5622 resulted in sex-specific behavior effects, with female mice developing long-term hyperactivity and anxiolytic-like behavior (46).

In the developing brain, microglia shape neural circuitry by: 1) inducing neuronal cell death *via* the release of superoxide ions (53, 54), 2) clearing viable (55) and apoptotic neural progenitors (56), 3) promoting neurogenesis *via* the release of IL-1β, IL-6, TNF, and IFN-γ (57–60), and 4) paring down supernumerary synapses, whilst strengthening functional ones (61–63). A number of mechanisms have been identified which contribute to microglial-meditated synapse modulation. Complement cascade components, C1q and C3, localized to neuronal synapses, promote microglial synapse engulfment (60, 64), while CD47 localized to neurons provides a “don’t eat me signal” to microglia that express CD172a (SIRPα), thereby preventing aberrant synaptic phagocytosis (65). Serotonin signaling (66), triggering receptor expressed on myeloid cells 2 (TREM2)-dependent functions (67), the CX3CR1-CX3CL1 axis (62) and microglial interaction with neuronal-expressed major histocompatibility complex (MHC) class I (68–71) are also thought to be involved in microglial-mediated synapse elimination. Microglia express CX3CR1 (62), TREM2 (67), and a serotonin receptor (5-HT2B) (66), with the latter enabling their movement towards serotonin. Knockout of these receptors results in defects in synaptic refinement (CX3CR1 and TREM2) or the organization of retinal projections (5-HT2B). Although microglia can prune superfluous synapses, they can also promote the formation of new ones (37, 72).

Beyond shaping neuronal circuitry, microglia are also required for vascularization, myelination, and gliogenesis. Microglia are recruited to growing vessels to promote vascular network formation in the retina (73, 74) and this is *via* release of angiogenic factors other than vascular endothelial growth factor–A (74). CD11c^+^ microglia, which expand in the postnatal brain, express a neurosupportive gene signature and IGF-1 and are required for myelinogenesis during development (75). More recently, a new role in gliogenesis has been identified for microglia at the later embryonic stages of E15.5 and E17.5 (76). A subpopulation of amoeboid microglia lining the tuberal hypothalamic third ventricle have been found to influence glial precursors *via* chemokine signaling, namely, CCL2 and CXCL10, which are required for the migration and maturation of oligodendrocytes, but not astrocytes (76). An additional unique microglial subset (*i.e*. proliferative-region associated microglia or PAM) enriched in metabolic genes and found in the first post-natal week in the corpus callosum and cerebellar white matter were found to be specialized in the clearance of newly formed oligodendrocytes (77).

### Adult Brain

In the adult brain, microglia tile the parenchyma in a grid-like fashion, displaying a ramified morphology with static somata and “never-resting” cytoplasmic extensions (78). These extensions survey the CNS microenvironment using their “sensome” to identify and respond to perturbations that may threaten homeostasis (79). The TWIK-related Halothane-Inhibited K^+^ channel, a tonically active potassium channel expressed by microglia, regulates the ramification and movement of microglial processes to support homeostatic surveillance of CNS activity (80). The microglial “sensome” comprises microglia-expressed genes encoding receptors and signaling molecules that enable detection of pathogen invasion, cytokines, pH alterations, metabolites, ATP, and adenosine. These include toll-like receptors (*Tlr2* and *Tlr7*), chemokine receptors (*Ccr5*, *Cx3cr1*, *Cxcr4*, and *Cxcr2*), Interferon-induced transmembrane proteins (*Ifitm2*, *Ifitm3*, and *Ifitm6*), Fc receptors (*Fcer1g* and *Fcgr3*), siglecs (*SiglecH* and *Siglec3/Cd33*), and purinergic receptors (*P2rx4*, *P2rx7*, *P2ry12*, *P2ry13*, and *P2ry6*) (79). P2RY12 and SiglecH are microglia-specific in the CNS, with P2RY12 importantly involved in chemotaxis towards neuronal and CNS damage *via* the detection of ATP or ADP (81, 82). In the aging brain, 81% of these genes are downregulated, with some genes, including *Cxcr4*, *Cxcr2*, *Tlr2*, *Ifitm2*, *Ifitm3*, *Ifitm6*, and *P2rx4*, being upregulated (79). This is thought to contribute to age-related microglial neurotoxicity (79) and potentially reduced microglial phagocytic activity that occurs with aging (83). Microglia also display an increased expression of CD11b, MHC-II, CD68, and CD86 proteins and expression of *Tnf*, *Il-6*, and *Il-1β* RNA in the aging brain, collectively suggesting an enhanced inflammatory profile and reduced homeostatic function with age (84).

The maintenance of a surveillant microglial state under physiological conditions is ultimately likely to be a vectorial outcome of a number of signals, including neuronal and astrocyte-derived factors, microglia-expressed CX3CR1, CD200 receptor (CD200R), and CD172a, which dampen microglial activity through binding their respective ligands, CX3CL1 (expressed by neurons), CD200 (expressed by neurons, astrocytes, and oligodendrocytes), and CD47 (expressed ubiquitously, including on neurons) (85), as well as through increased expression of microRNA-124 (86) and TGF-β signaling (25).

Besides tissue surveillance, microglia are involved in synapse formation and learning in the adult CNS *via* the secretion of brain-derived neurotropic factor (37). Microglia are also required for synaptic pruning, with the purine receptor P2RY12 important for synaptic plasticity in the visual cortex of the adolescent CNS (87). In contrast, the CX3CR1-CX3CL1 (62) and CR3/CD11b (60) axis appear to be more critical during development for microglial-mediated synaptic pruning. Microglia also support adult neurogenesis, with a unique population of microglia expressing low levels of purine receptors in the subventricular zone and rostral migratory stream required for survival and migration of newly generated neuroblasts (88).

The role of microglia as phagocytes also plays a major part in homeostasis, enabling clearance of debris, apoptotic, and surplus cells (89) to maintain optimal neural function. Microglial-expressed TAM receptor kinases, MER Proto-Oncogene Tyrosine Kinase (MerTK), and Axl have revealed an important role for neuronal progenitor cell clearance (90), which may be required for efficient neurogenesis, whereas CD11b, TREM2, TIM-4, and BAI1 appear to be required for the phagocytosis of apoptotic neurons (53, 91, 92). Microglia can recognize a number of “eat-me” signals, including phosphatidylserine, components of the complement system, thrombospondin and uridine 5′-diphosphate (93), which stimulate phagocytosis (68, 76, 93). Although microglia are the principle phagocytes in the CNS, other glia, including oligodendrocytes and astrocytes, are also thought to contribute to this function (94, 95).

## Microglial “Activation”

Microglial “activation” refers to a reversible, transient state, defined by a morphological and functional phenotype distinct from homeostatic microglia. Before the advent of *in vivo* imaging, microglia in steady state homeostasis were classified as “resting.” However, it is now clear that although the cell soma may remain in one site, the processes of each microglia continuously explore the microenvironment in a highly dynamic manner (78).

In the steady state, microglia have a small cell soma with long, thin hyper-ramified cytoplasmic processes. On detection of a noxious signal (toxins, pathogens, endogenous proteins) or neuronal damage, microglia undergo a rapid morphological transition, retracting their processes to become shorter and thicker, acquiring a more ameboid morphology, and undergoing hypertrophy, thus increasing their somatic surface area. In addition to these morphological adaptations, often referred to as microgliosis, microglia undergo transcriptional and phenotypic changes in a context-dependent manner. This reactive phenotype is associated with changes to motile, proliferative, and phagocytic functions (96, 97) and invites comparison with microglia that populate the early CNS. Historically, alterations in microglial morphology and/or the upregulation of CD45, Iba1, Griffonia *simplicifolia*-lectin, and MHC-II were the first reliable indicators of microglial “activation” that implicated microglia in CNS pathology.

Intermediate morphological activated states of microglia have also been identified, which are described as “rod-like,” “hyper-ramified,” “bi-polar,” and “bushy” (98). However, it is clear that microglial form and function do not necessarily correspond, as microglia are observed to display both classic “resting” and “activated” morphologies in human CNS inflammation and neurological and psychiatric disease (99). Despite morphological measurements (cell somatic area, dendrite length and number, total cell area, and parenchymal cell density) being the primary technique used to study these cells for decades, there are no standard parameters that link these forms to function and more detailed *in situ* molecular and protein profiling techniques, paired with imaging will be required to fill this gap. The Hyperion is an imaging mass cytometer and one of the first multiplexed imaging technologies developed which theoretically enables the detection of >100 different metal-conjugated markers (currently 49) to enable spatial resolution of protein expression in tissue sections (100). Other competing high-dimensional imaging systems include the CODEX, GeoMx DSP, and the MACSima by Akoya Biosciences, Nanostring, and Miltenyi Biotec, respectively. To fully recapitulate the dynamic nature of these cells in tissues in real-time, *in vivo* imaging techniques, such as intravital microscopy (IVM) can be employed. However, with the limited number of fluorescent probes and mouse models available for IVM, correlative imaging, combining data from fluorescence, light and electron microscopic modalities provide additional structure-function information (101, 102).

Advances in high-dimensional and single-cell molecular and immune profiling technologies have effectively invalidated classical microglial characterization approaches. The descriptive “resting” *versus* “activated” and “M1” *versus* “M2” nomenclature oversimplified microglial behavior, suggesting they exhibited dichotomous “yin-yang”-like functions. These concepts have been rejected by the field (103) and are being replaced by multi-dimensional activation states, in which function is programmed and then finely tuned according to the prevailing microenvironment, in a context-, sex-, region-, developmental-, disease-, and even disease stage-specific manner. It is still accepted that microglia have pro-inflammatory (“M1”) and anti-inflammatory (“M2”) functions, but these are now understood to co-exist, with microglia capable of co-expressing M1-like and M2-like markers in a context-dependent manner. Thus “disease-associated” microglia (DAMs) in a mouse model of Alzheimer’s disease (104), “microglial neurodegenerative” phenotype (MGnD) in mouse models of AD and ALS (105) and disease-associated microglia (daMG1-4) in experimental autoimmune encephalomyelitis (EAE) (106) are superseding earlier and more simplistic terms, to incorporate the idea that microglia can have unique molecular and/or immunological profiles and/or functions in different disease contexts.

## Origin and Classification of Monocytes and Monocyte-Derived Cells

During certain diseases and/or injuries involving breach of the BBB, BM-derived monocytes infiltrate the CNS parenchyma and intermingle with the resident microglial population. Despite often close phenotypic similarities, these infiltrating myeloid cells are developmentally distinct from microglia and give rise to effector cells whose functions are presumably not fulfilled by their resident counterparts. In contrast to the YS-origin of microglia, monocytes are hematopoietic cells that originate in the BM. In adulthood, these cells are derived from definitive HSC and mature from monocyte-dendritic cell (MDP) precursors, common monocyte progenitors (cMoP), and granulocyte and macrophage progenitors (GMP) through a series of sequential differentiation steps in the BM (107, 108). The fate of these monocytes is specified by the expression of transcription factors PU.1, IRF8, Klf4, and GATA2 (3, 109–111), and their differentiation, survival, and proliferation is regulated by the growth factor receptor CD115 and its ligand M-CSF (112–114). Following their generation in the BM, monocytes are released into the peripheral circulation.

Circulating monocytes are composed of multiple subsets that differ in their phenotype, size, transcriptional profiles, and migratory properties. These distinct monocyte subsets are characterized by their differential expression of CD14 and CD16 in humans (115) and by the surface marker combination Ly6C, CD62L, CD43, and the chemokine receptors CX3CR1 and C-C chemokine receptor 2 (CCR2) in mice (116) (**Figure 1**). In humans, 80–90% of the monocyte pool is composed of CD14^+^CD16^−^ classical monocytes with the remaining 10–20% shared by CD14^+^CD16^+^ intermediate and CD14^lo^CD16^+^ non-classical monocytes (115). The generation of a mouse strain in which a GFP reporter was engineered into the *CX3CR1* locus (*CX3CR1* ^GFP^ mice) (117) enabled the discovery of two corresponding monocyte subsets (116). In mice, “classical” monocytes (also known as “inflammatory monocytes”) are characterized by their expression of surface markers Ly6C^hi^, CX3CR1^int,^ CCR2^+^, CD62L^+^, and CD43^lo^, whereas “non-classical” monocytes (also referred to as “patrolling monocytes”) are defined as Ly6C^lo^, CX3CR1^hi^, CCR2^lo^, CD62L^−^, and CD43^+^ cells (116, 118, 119). Transcriptional comparison between mouse and human monocyte subsets correlated Ly6C^hi^ monocytes with classical CD14^+^CD16^-^ monocytes and Ly6C^lo^ monocytes with non-classical CD14^lo^CD16^+^ monocytes (120).

As a component of the mononuclear phagocyte system, circulating monocytes were historically considered to be the definitive precursors of tissue-resident macrophages and dendritic cells (DC) (121). However, recent studies have demonstrated that most tissue-resident macrophages are of embryonic origin (122, 123), although conventional DCs have a distinct BM precursor (124). Today, monocytes are viewed as a distinctive cell type with diverse functions. In the steady state, Ly6C^+^ monocytes can traffic to various tissues and maintain their monocytic transcriptional profile (119), but they can also give rise to a proportion of tissue-resident myeloid cells (123) or transition into Ly6C^lo^ monocytes (123, 125, 126). During inflammation, monocytes can give rise to macrophages (monocyte-derived macrophages or MDMs) and DCs (monocyte-derived DC or moDCs) with non-redundant functions that often cannot be fulfilled by their resident counterparts (126). Collectively, these distinctive cell types have been classified according to their monocytic origin as “monocyte-derived cells” (MDC) (127) (**Figure 1**).

Under homeostatic conditions, Ly6C^hi^ monocyte progeny are present in almost all tissues, where they constitute a minor fraction of the tissue-resident macrophage pool (119, 128–134). The CNS parenchyma is a notable exception, where little to no monocyte immigration is observed in the steady state (5, 12, 135), although a proportion of choroid plexus and dural macrophages are evidently replenished by BM-derived monocytes during homeostasis (15, 136).

## Ontogeny and Differentiation of Monocyte-Derived Cells in the Inflamed CNS

In contrast to homeostasis, during inflammation Ly6C^hi^ monocytes may rapidly infiltrate the diseased CNS, usually in a CCR2-dependent manner. This may be facilitated by compromise of the BBB, but not necessarily (30, 137). Although monocyte recruitment and infiltration is well described in the acutely diseased brain, the behavior of these cells is more controversial in chronic, low grade inflammation observed in aging and stress. Thus, despite increased BBB permeability with age, monocyte infiltration does not inevitably accompany healthy aging (138). On the other hand, inflammation associated with psychosocial stress may promote monocyte infiltration into the CNS (139, 140), although this has been contested (141, 142).

Interactions between monocytes and CNS borders critically affect their recruitment, infiltration, and differentiation during neuroinflammation. The different ports of entry into the CNS have been implicated in shaping either a protective or pathogenic monocyte response. For instance, the differential expression of CX3CR1 and CCR2 ligands may selectively recruit either “pro-inflammatory” (Ly6C^hi^CCR2^+^) or “pro-resolution” (Ly6C^lo^CX3CR1^hi^) monocyte-derived cells. This is supported by experiments showing that Ly6C^lo^CX3CR1^hi^ monocytes, which aid recovery from spinal cord injury, entered the CNS *via* the choroid plexus and migrated to the injury site through the central canal in an α4-integrin/vascular cell adhesion molecule-1- and CD73-dependent manner. In contrast, Ly6C^hi^CCR2^+^ pro-inflammatory monocytes entered the CNS *via* the parenchymal blood vasculature in a CCL2-dependent manner and mediated secondary injury (143). Although Ly6C^hi^ and Ly6C^lo^ monocytes are thought to be independently recruited to the CNS, the transition of Ly6C^hi^ monocytes to Ly6C^lo^ monocytes has been observed during both homeostasis and inflammation, and the recruitment of Ly6C^lo^ monocytes is at least partially CCR2-dependent (123, 125, 126). It is possible that the transition from Ly6C^hi^ to Ly6C^lo^ monocytes is influenced by different CNS entry points, such that monocytes traversing through choroid plexus and leptomeninges encounter stimuli driving their differentiation into Ly6C^lo^ monocytes, whereas those traversing through the parenchymal vasculature remain undifferentiated inflammatory monocytes. Alternatively, the endothelium may better enable the emigration of Ly6C^hi^ cells from the CNS parenchymal vasculature (144). Future studies investigating how endogenous macrophages and/or endothelium at various CNS-entry points may shape the phenotypic and functional profiles of CNS-infiltrating Ly6C^hi^ monocytes in the mature animal are needed to address these gaps. Furthermore, what changes occur during development of the BBB that enable differential diapedesis during maturation of the adaptive immune system have yet to be fully elucidated.

Once in the CNS parenchyma, local microenvironmental cues can shape MDMs to adopt a phenotype similar to those of CNS-resident macrophages. Using CCR2-red fluorescent reporter (RFP) mice, a recent study found CNS-infiltrating CCR2^+^CD206^+^ monocyte-derived cells localized beside CCR2^-^CD206^+^ resident macrophages in the leptomeninges and perivascular space, demonstrating these cells can gain phenotypic markers characteristic of CNS-resident myeloid cells (106). Similarly, CNS-infiltrating monocytes adopt a phenotype indistinguishable from microglia in the acute phase of EAE, although these cells do not appear to integrate into the CNS-resident microglia population following the resolution of inflammation (145, 146).

Emergency conditions may additionally generate ontogenically distinct monocyte subsets whose presence is restricted to inflammatory conditions. As severe inflammation requires the constant generation and mobilization of monocytes to the inflamed brain, emergency monopoiesis can generate GMP-, MDP-, and cMoP-derived monocytes that appear under inflammatory conditions (108) and may perhaps bypass the canonical Ly6C^+^ monocyte intermediate (147). In the inflamed brain, such populations may include *Cxcl10* ^+^ and *Saa3* ^+^ monocytes, the former having been identified in EAE and possibly cerebral malaria (147, 148). Whether these “emergency” monocyte populations are functionally distinct from Ly6C^hi^ monocyte-derived cells is unclear, although recent evidence suggests these subsets may differentially contribute to pathology (147). Further fate-mapping and functional studies investigating emergency monocyte populations in the inflamed CNS will be needed to assess whether these cells are ontogenically and functionally distinct from those derived from Ly6C^hi^ monocytes during neuroinflammation. Taken together, monocytes represent a particular unique, plastic cell type equipped with a diverse differential program that enables their context-dependent effector functions upon entry into the CNS.

## Identifying Microglia in the Homoeostatic and Inflamed Brain

Studying microglial behavior in the brain is difficult, even under homeostatic conditions. Separating microglial functions from other neuroglial or peripherally derived immune cell responses is challenged firstly by the difficulty of culturing adult murine microglia (149) and secondly, by their tendency to alter their transcriptome *ex vivo*. Human and mouse microglia lose their *in vivo* transcriptional profile upon isolation, with significant differences in mRNA signatures between recently isolated microglia and *in vitro*-cultured microglia (25, 150), although the *in vivo* profile may be restored when cells are put back into an intact brain (151). This emphasizes the likely need for interaction with other CNS cell types for “normalcy” and it is likely that loss of environmental cues remodel the regulatory milieu *in vitro*, inducing substantial changes in microglial gene expression (150). Culturing mouse and human microglia for only 6 h induced upregulation of genes related to acute inflammatory response and stress and downregulation of genes associated with immune functions, as well as blood vessel and brain development (150). Although culturing conditions required to maintain the *in vivo* microglial transcriptome are unknown, brain-specific signals are almost certainly required, currently limiting the interpretation of *in vitro* observations. Our growing understanding of the inextricable importance of the brain microenvironment in instructing microglial phenotype and behavior thus drives an increasing emphasis on work *in vivo*.

In the homeostatic brain, microglia are easily identifiable from other cells in the CNS (see **Table 2** for a list of microglial phenotypes identified in the adult murine brain in steady state). Microglia comprise the largest myeloid population in the CNS and can be identified using imaging or single-cell cytometry systems with one or two of a wide range of phenotypic and/or functional markers, *e.g*., CD45, CX3CR1, CD11b, F4/80, CD64, CD68, transmembrane protein 119 (TMEM119), purinergic receptor P2Y, G-protein-coupled 12 *(*P2RY12), CD115 (CSF-1R), CD200R, CD172a (SIRPα), CD317, MerTK, 4D4, lymphocyte antigen 86 (LY86), secreted protein acidic and rich in cysteine (SPARC), CD162, and Fc receptor-like S (FCRLS) (106, 136, 146, 153–157) (**Figure 1** and **Table 2**). Using flow, mass, and spectral cytometry, murine and human homeostatic microglia are typically identified as CD45^lo^CD11b^+^ (30, 154, 158). Non-parenchymal brain macrophages, *i.e.*, dural, meningeal, perivascular, and choroid plexus macrophages, collectively called CNS- or border-associated macrophages (CAMS or BAMS) (159–161), have a higher expression of CD45 (CD11b^+^CD45^int^) and/or do not express microglia-specific markers, making these cells distinguishable from microglia (136). By immunohistochemical techniques, microglia are commonly recognized by their immunoreactivity to Iba1, CD11b, CD68, and GS-lectin. Moreover, the highly ramified morphology of microglia makes them readily distinguishable from other myeloid cells in the brain, which are more amoeboid in shape (162).

**Table 2:** Genes and proteins expressed by microglia in steady state.

**Transcriptome**	**Two microglia subsets, hMG1 and hMG2, both expressing**: *Bhlhe41*, *Gpr34*, *Hexb*, *Olfml3*, *P2ry12*, *P2ry13*, *Sall1*, *Serpine2*, *Siglech*, *Sparc*, *Cx3cr1*, *Fcr1*, *Csfr*, *Csf1*, *C1qc*, *C1qb*, *C1qa*, *Tmem119*, *Trem2*, and *Slc2a5* (hMG1 express genes related to the ERK1 and ERK2 cascade as well as responses to IFN-γ) [Single-cell RNAseq, (106)]
	*Hexb*, *Cst3*, *Cx3cr1*, *Ctsd*, *Csf1r*, *Ctss*, *Sparc*, *Tmsb4x*, *P2ry12*, *C1qa*, and *C1qb* [Single-cell RNAseq, (104)]
	*Fcrls*, *Trem2*, *Hexb*, *Olfml3*, *Gpr34*, *Tmem119*, *P2ry12*, *Siglech*, *Golm1*, *Sall1*, *Adgrg1*, *Slc2a5*, *Serpine2*, *Sparc*, *Adamts1*, *Itgam*, *Aif1*, *Cx3cr1*, *Csf1r*, *Cd68*, *Adgre1*, *Fcgr1*, and *MerTK* [Single-cell RNAseq (136)]
**Proteome**	CD45^+^CD11b^+^F4/80^+^CD64^+^MerTK^+^CD24^+^ CD172a^+^ [CyTOF (152)].
	**Two microglia subsets, A and B, both expressing:** CD45^+^CD11b^+^CD317^+^MHC-II^-^CD88^+^MHCI^+^MerTK^+^4D4^+^FCRLS^+^ ***Unique expression profiles between the microglia subsets:*** **Pop A:** CD39^low^CD86^−^ **Pop B:** CD39^hi^CD86^+^ [CYTOF (146)]
	CD162^+^P2RY12^+^TMEM119^+^Ly86^+^Iba-1^+^ SPARC^+^ [IHC (106)]
	**Three microglia subsets, 1–3, all expressing:** CD45^low^CX3CR1^+^CD11b^+^F4/80^low/−^ ***Unique expression profiles between the three microglia subsets:*** **Subset 1:** CD14^+^TCR-β^+^ **Subset 2:** CXCR4^+^CCR5^+^CD115^+^ (Could represent a more motile population) **Subset 3:** MHCII^+^ (Could be of peripheral origin) [CyTOF (153)]a	CD45^+^CD64^hi^CD11c^low^MMR^low^MHCII^low^CD11b^hi^CLEC12A^low^ NRP1^low^CD63^low^[Flow cytometry (136)]

However, identification of microglia using immunohistochemistry or cytometry becomes increasingly complicated during neuroinflammation with the infiltration of BM-derived monocytes that adopt a phenotype and morphology similar to reactive microglia. Infiltration of MDMs into the CNS is a hallmark of a number of acute and chronic neuropathologies, including autoimmunity, neurodegeneration, stroke, traumatic injury, and infection, with each disease context associated with a varying degree of CNS infiltration, inflammation, as well as differential MDM and microglial phenotype and function.

CNS-infiltrating MDMs express molecular markers common to microglia, including CX3CR1, CD11b, F4/80, CD45, CD64, CD115, and Iba1, to name a few (154). On the other hand, these cells express higher amounts of Ly6C, CD44, CD45, CD49d, CD11a, CXCR4, and CCR2 and have a lower expression of CX3CR1 (30, 153, 154, 163–165). These markers, however, can be downregulated over the course of disease. Typically, MDMs are identified as CD11b^+^CD45^hi^. However, since BAMs are also CD45^int/hi^ and “activated” microglia upregulate CD45, this gating system fails to accurately discriminate between these cells. This is particularly true in severe inflammatory conditions, such as West Nile virus (WNV) encephalitis, where there is substantial and sustained infiltration of MDMs into the CNS (30). Thus, the ability to resolve populations during neuroinflammation has historically been impossible without recourse to adoptive transfers, parabiosis, or chimeric animals made by lethal gamma-irradiation and BM reconstitution. Although identification of resident and infiltrating cells becomes clearer using such techniques, the non-physiological conditions may confound the accurate interpretation of results.

## Tools Used to Discriminate Resident and Infiltrating Myeloid Cells in the Inflamed Brain

Recent advances in single-cell sequencing technologies has shed light on some uniquely expressed microglial genes including *Fcrls*, *P2ry12* (25), *Spalt-like transcription factor 1* (*SALL1*) (38), *sialic acid-binding immunoglobulin-type lectin H* (*Siglec-H*), and *Tmem119* (166). The development of RNA primers and antibodies against these “microglia-specific” markers have substantially aided in the resolution of myeloid populations in the CNS, without the need for more complicated experimental manipulation. Transgenic animals expressing fluorescent reporters that identify microglia, or Cre-recombinase and/or HSV-TK under “microglia-specific” promoters that can be used to deplete microglia, have also been an important advance on the use of CX3CR1 or CD11b promoters, which also act on myeloid cells in the periphery.

However, the discovery that microglia-specific markers P2RY12 and TMEM119 are downregulated in neurodegeneration and neuroinflammation (105, 106, 167), has reduced their value for identification of microglia in such models. Nonetheless, the expression of these markers appears to be model-dependent and therefore more useful in specific diseased-states. P2RY12 was upregulated in models of pseudorabies virus encephalitis (168) and neuropathic pain (169), whilst P2RY12 (170) and TMEM119 (163, 171) were stably expressed during stroke. However, both markers have been shown to be expressed by peripherally-derived myeloid cells in the CNS (9, 163), with TMEM119 also expressed by other non-CNS cell types (172). TMEM119, originally shown to be expressed in mouse osteoblasts, is additionally expressed in human bone tissue, DCs, osteosarcoma, and lymphoid tissue (173, 174). FCRLS, also previously thought to be microglia-specific, has been observed in all CNS-associated macrophage subsets (106). Notwithstanding these limitations, these markers are still specific for microglia in the homeostatic CNS and will likely remain important tools for elucidating function.

Another major advance in microglial biology has been the discovery of PLX5622 (Plexxikon Inc.) (21), a small molecule CD115 inhibitor that penetrates the BBB and depletes microglia in as little as three days (175, 176) (**Table 1**). Other studies have reported near to complete microglial depletion within 7, 14, or 21 days. Not surprisingly, other cells dependent on CD115 signaling are also modulated by PLX5622 treatment, including lymphocytes and myeloid cells in the spleen, blood and BM (42). Moreover, some microglia are resistant to depletion even after prolonged treatment, making this approach unsuitable for studying all microglia subtypes (21). Despite these limitations, PLX5622 is a major improvement from previously used depletion methods including i.c.v.-injected clodronate liposomes, PLX3397 (also a CD115 inhibitor), CD11b-HSVTK, and *CX3CR1* ^CreER^DTR mice, all of which may non-specifically target other leukocytes, with some methods taking longer for microglial ablation to occur or associated with incomplete microglial depletion and/or toxicity, off-target effects, or BBB damage (34) (**Table 1**). Moreover, PLX5622, unlike PLX3397, has a 20-fold greater selectivity for CD115 than for other kinases, as well as increased BBB penetration (21).

Although PLX5622 has become the gold standard microglial depletion method, CNS changes that subsequently occur in the absence of microglia and/or in the presence of dead microglia, limit the accurate interpretation of their cellular functions *in vivo*. *In vivo* fate-mapping models used to track peripheral or resident cells have largely overcome this limitation. The development of site-specific recombinases and transgenic mice, for instance, have provided tools to genetically mark cell lineages and their descendants, enabling the mapping of cell interaction and migration, lineage segregation and proliferation (177–179). Thus, unlike the aforementioned methodologies used to study microglial functions, fate-mapping provides a targeted and non-invasive approach that can be used during development and adulthood. Further, in contrast to conventional reporter strains whereby mice express fluorescent reporters under specific promoters (e.g. CX3CR1^GFP/+^ or CCR2 ^GFP/+^ or CX3CR1^GFP/+^; CCR2 ^GFP/+^ mice), fate-mapping does not require markers to be stably expressed by cells. Thus, enabling the identification of cells following the downregulation of relevant genetic markers. Fate-mapping approaches have been used in a number of neuroinflammatory models to distinguish resident from infiltrating myeloid cells (9, 163). For example, using *Cxcr4* ^CreER/Wt^ *; R26* ^CAG-LSL-tdT^ mice in a stroke model, HSC-derived myeloid cells were traceable by tdTomato (tdT) fluorescence (163). Moreover, the ubiquitously active CAG promoter in *R26* ^CAG-LSL-tdT^ enabled MDMs to be traced, despite their downregulation of CXCR4 in the CNS during stroke. A similar approach was used in neonatal stroke and development using bi-transgenic CCR2-CreER^tg/+^; R26R-EGFP^tg/+^ mice, where Ly6C^hi^ and Ly6C^lo^ cells could be mapped despite downregulation of CCR2 (9). Although fate-mapping is a powerful approach that can be used to study microglial functions *in-vivo*, these models can be time-consuming and costly to generate, as well as requiring cell-specific markers to target particular cell types.

The development of high-parameter cytometry systems, including mass and spectral cytometry have further aided the necessary discrimination of populations without genetic manipulation. With a generally enhanced signal sensitivity, spectral cytometers such as the Cytek^®^ Aurora can enable more accurate separation of cells which may differ in their relative expression of single and/or dim markers. The ability to measure a greater number of fluorescent signals in one assay and the speed of acquisition gives spectral cytometry a significant advantage over conventional fluorescence flow and mass cytometry (180). Nevertheless, high-dimensional immune profiling by these modalities, in conjunction with dimensionality-reduction algorithms, such as t-distributed stochastic neighbor embedding (tSNE) and uniform manifold approximation (UMAP), which enable the visualization of high-dimensional data on a 2D plot, provides important tools for more detailed population identification and separation (181–184). The use of unbiased clustering and dimensionality-reduction approaches assist in the identification of subpopulations with a range of differentially expressed markers. The development of novel gating strategies arising from this separation further enable cell types to be sorted for more detailed *in vitro* or *in vivo* functional or RNA analysis. In EAE, for instance, three microglial subpopulations were identified with mass cytometry by two independent groups (106, 146), with one of these studies also identifying five MDM subsets (146). Understanding the protective or pathogenic functions of these cell types will inform targeted cell-specific therapies in these diseases. Taken together, the development of new tools to resolve myeloid populations in the CNS has substantially enhanced our understanding of their functions and heterogeneity in health and disease.

## Shoes Too Big to Fill? Can Monocyte-Derived Macrophages Acquire a Microglial Identity?

Considering microglia seed the brain during early embryogenesis, where they participate in CNS development, support neuronal networks, and adopt memory-like functions as they persist throughout adulthood, is it possible for MDMs, with a different origin, epigenome, and transcriptome, to acquire a “true” or even a functional microglial identity? Similar to microglia, tissue-resident Kupffer cells in the liver and alveolar cells in the lung are established before birth and are subsequently renewed in situ independently of BM-derived monocytes (7, 132). However, monocytes show minimal transcriptomic differences with their embryonic counterparts and can differentiate into both Kupffer cells and alveolar macrophages (185–187), but evidently not into microglia. Peripheral monocytes can populate the CNS, but they differ phenotypically, have a non-redundant role and a different molecular signature from embryonically seeded microglia (25, 188). Even after prolonged engraftment in the brain, MDM responses to lipopolysaccharide challenge, chromatin landscapes and ~2000 transcripts remained different from resident microglia (189). Engrafted MDMs did, however, adopt other microglial characteristics including self-renewal, resistance to γ-irradiation and a ramified morphology (190). In contrast, donor microglial cells fully adopt the transcriptomic identity of embryonically derived microglia in microglia-deficient CD115 knockout mice (191). Why BM-derived myeloid cells only become “microglia-like” in the CNS is currently unknown, but the EMP origin of microglia and the unique CNS tissue microenvironment likely plays a critical role (150, 191).

In contrast, a small population of microglia are reported to be derived from BM-derived HSCs during embryogenesis, suggesting a monocyte to microglia switch (9). This has also been demonstrated during neonatal stroke using a fate-mapping model, where invading monocytes became DCs or microglia-like cells (9). Microglia-like cells were present 62 days post-stroke, with many exhibiting a ramified morphology, P2RY12 and TMEM119 immunopositivity and expression of *Sall1* mRNA. In another stroke model, MDMs ectopically placed in the peri infarct region of *Cxcr4* knockout mice became positive for P2RY12 and TMEM119 (163). In WNV encephalitis models, Ly6C^hi^ monocytes migrate from the BM to the CNS, where they assume a phenotype indistinguishable from activated microglia, with regard to CD45 and CD11b expression (30, 192). Contrary to the view that microglia-like cells enter the brain only when the BBB is perturbed, the BBB is only sporadically affected in this model (30). Some of these peripherally derived monocytes also became ramified in the parenchyma of the brain (30).

Further investigation is required to understand why infiltrating MDMs express microglial molecules in the CNS *de novo*, and the putative functions of these peripherally derived cells, relative to their resident counterparts. It is possible that TMEM119 and P2RY12 are not microglia-specific in the inflamed CNS, or that the inflammatory milieu in stroke, coupled with the prolonged time MDMs spend in the CNS, enables them to acquire a microglia-like phenotype, particularly as the CNS microenvironment ordinarily defines microglial phenotype and identity (150, 191). The degree of inflammation may be important; WNV causes a fatal encephalitis characterized by severe inflammatory monocyte infiltration that involves the entire CNS (30), whereas models such as EAE or AD, used to investigate microglial activity, are accompanied only by localized foci of inflammation and/or much less severe inflammation overall. As such, the response observed in WNV may be in stark contrast to what has previously been described. It is worth reflecting from an evolutionary point of view that the biggest threat to survival is infection, against which the best defense is the primed innate and adaptive immune systems. Long-lived animals are subject to many infections over a lifetime, as well as having an environmentally increased probability of being infected by the same pathogen more than once. As such, it seems reasonable that myeloid reservoirs in the BM compartment could be recruited to the brain to perform microglial functions in the interim. Setting up novel “microglial” networks during a first CNS infection in a high prevalence environment, despite a possible functional cost, may be a useful survival strategy for effective early CNS defense by the innate immune response in the event of novel or recurring future infections. Irrespective of whether MDMs can become microglia physiologically, current approaches are being developed with the intention of engineering these cells for therapeutic use in CNS disease and will undoubtedly yield further insight into the developmental plasticity and range of functions in this lineage, as well as providing additional investigative tools for ongoing study.

Therapeutic ablation of microglia in AD and ALS, where microglial activity has been shown to enhance disease severity, has been proposed in conjunction with engraftment of adoptively transferred myeloid cells (193). However, knowing whether transferred myeloid cells will contribute to undesirable or unexpected adverse effects, due to their inability to mimic microglial behavior and perhaps fulfil microglial homeostatic roles, would be important to know. Other studies have attempted to generate microglia from human induced pluripotent stem cells in a defined media (194–197) to study human microglial behavior as well as for therapeutic prospects. Methods used to generate microglia are reviewed elsewhere (198). However, mRNA analysis showed that microglia from human induced pluripotent stem cells exhibited a phenotype similar to *in vitro* microglia rather than *ex vivo* microglia (150). More complex culturing conditions may be required to induce and maintain a microglial phenotype, including the use of organoids and co-culturing with glial cells (including astrocytes and oligodendrocytes). Understanding the specific gene-environment interactions that shape microglial phenotypes in different contexts will help inform ways to generate “microglia” as well as revealing what influences their phenotypic switch during disease. More recently, the development of human pluripotent stem cell (hPSC)-based microglia chimeric mouse brains, in which hPSC-derived cells are engrafted into neonatal mice, has evidently overcome the limitations of using cultured microglia to study these cells (199). Single-cell RNA sequencing data showed that these xenografted microglial cells resembled human microglia. Considering species-specific differences between microglia in humans and mice (150), this model provides a unique opportunity to study the role of human microglia in the intact brain.

Microglia, once considered a bystander of CNS physiology and pathology, are now in the spotlight of neuroimmune research. Single-cell protein and RNA sequencing technologies, *in-vivo* imaging and lineage-tracing techniques have substantially improved the delineation of myeloid populations in the CNS, as well as, our understanding of microglial physiology, ontogeny, and heterogeneity. This will likely elucidate their disease-related functions and inform targeted therapeutics.

## Author Contributions

AS, CW, and NK all contributed to the writing and conceptualization. CW was responsible for illustrating **Figure 1**. All authors contributed to the article and approved the submitted version.

## Funding

This work was supported by funding from the Merridew Foundation and NH & MRC Project Grant 1088242 to NK. AS is supported by the Australian Government Research Training Stipend Scholarship and The University of Sydney Postgraduate Merit Award.

## Conflict of Interest

The authors declare that the research was conducted in the absence of any commercial or financial relationships that could be construed as a potential conflict of interest.

The handling editor declared a past collaboration with one of the authors, NK.
